# Photophobia, Resulting from Exalted Sensibility of the Sensitive Branch of the Fifth Pair Going to the Eye from Irritation of the Sensitive Branch of the Same Nerve Going to the Teeth

**Published:** 1849-09

**Authors:** 


*Photophobia, resulting from exalted sensibility of the sensitive branch of the fifth pair going to the eye from irritation of the sensitive branch of the same nerve going to the teeth.*—Dr. Hays remarked, that he had seen, within a few years, some curious cases of exalted sensibility of the retina, from a cause which he believes has not been suspected of such an effect, viz: irritation of the dental branch of the fifth pair of nerves. These cases much interested him, and if the college had nothing better to occupy their attention, he would present a verbal sketch of a few of them; he had not prepared any written history of the cases, having no previous intention of submitting them at this meeting.

The first case occurred in a gentleman, the cashier of a bank in North Carolina. At the great fire in Wilmington, he had suffered considerable fatigue and exposure in endeavoring to save the books and papers of the bank, and had, subsequently, severely tried his eyes in arranging the documents which were rescued from the flames. He soon experienced great intolerance of light, and some inflammation of the conjunctiva. For this he was treated by the physicians in his vicinity, but with only temporary relief, except for the inflammation. He subsequently visited Virginia, and Raleigh, North Carolina, for medical advice; but from none of the remedies or plans of treatment employed in his case, did he experience the slightest permanent benefit; on the contrary, the photophobia increased to such a degree as to render exposure to the least light perfect torture. Dr. Hays was written to. Believing the case to be one for which it was not possible to prescribe judiciously until he was enabled to make a thorough examination of it, he requested that the gentleman should be brought on to Philadelphia; but his friends, in reply, stated that this would scarcely be possible, in consequence of the excessive photophobia under which the patient labored, rendering the slightest degree of light intolerable.

Dr. Hays suggested that the eyes should be entirely defended from the access of light by covering them with a mass of wadded silk. This suggestion was adopted.

When the gentleman reached the city, Dr. H. found him laboring under the most aggravated degree of photophobia. In a room so perfectly dark that the Doctor was unable to see any object whatever, to the patient the light reflected from his own hands was intolerable, and that from his shirt bosom caused so much suffering that he was obliged to keep the latter constantly covered. The colored nurse, whom he had brought on to attend upon him, happening to enter the darkened room, the light from a white apron she wore produced the utmost suffering to the patient. So exalted was the sensibility of the retina, that, in the darkened room, where Dr. H. could not see his hand held up before him, the patient was able to distinguish the objects around him, even the figures in the carpet.

He was, at length, persuaded to submit to an examination of his eyes, which he bore with great fortitude.

Dr. Hays found scarcely a trace of inflammation of the eyes or of any other apparent disease. The stomach of the patient was somewhat deranged. This being remedied without relief to the photophobia, the doctor was induced to seek for some other source of irritation, and, after careful examination of the patient, he was induced to suspect that the teeth, several of which were defective, but not painful, might be the source of the evil. At his suggestion, a couple were extracted by a dentist, but without causing any diminution of the intolerance of light. After some eight or ten days, Dr. H. examined the patient's mouth himself, and upon striking one of the lateral upper incisors nearest to the eye most affected, with a key, the patient winced as from pain, and stated that he had often experienced a disagreeable sensation to proceed from that tooth. The tooth was extracted ; with the loss of the tooth, a most disagreeable gnawing or pinching sensation at the back of the eye, which had previously tormented the patient, ceased. At the root of the tooth, there was found a large abscess, while the periosteum of the alveole was thickened. From this time the morbid sensibility of the eyes rapidly diminished, and the patient was soon after sufficiently recovered to return home and resume his duties as cashier of the bank. When Dr. Hays last heard from him, which was the past summer, after an interval of nearly six years, he was perfectly well, having had no return of the photophobia.

The next case was one which Dr. H. saw last fall, in consultation with his friend Dr. Ashmead, in a Spanish gentleman, who had suffered two years previously from a slight attack of iritis. On recovering from this, he experienced, whenever he attempted to read, a peculiar uneasiness in his eyes. For this, he consulted Dr. Ashmead, who advised a voyage to New Orleans ; finding no diminution in the affection of the eye, he proceeded from New Orleans to Cuba. The uneasiness still continuing unrelieved, he returned last fall to Philadelphia. Dr. H. now saw him with Dr. Ashmead, Upon examining the eyes, they were found to be without any trace of inflammation or other apparent disease. Still, whenever the patient attempted to read, the same uneasiness, which had now continued for some eighteen months, was experienced.

Judging from the circumstances of the case first detailed, Dr. H. was led to suspect that the source of the affection of the eye in the present case might be the same ; he accordingly examined the patient's teeth, from which, according to his account, he had experienced no suffering. Finding some of the teeth diseased, the Doctor directed them to be extracted. Only one was taken out, when the patient's courage failed him, and no relief was afforded to the affection of the eye. Subsequently, another tooth was extracted, at the root of which an abscess was found to exist. The patient now declared himself entirely relieved from the uneasy sensation he had so long experienced in his eyes on attempting to read. He has since continued perfectly well ; now eighteen months.

Another case occurred in a lady, marked by the same intolerance of light as in the case first described; in which Dr. Hays had every reason to believe that the morbid sensibility of the retina was produced by irritation of the dental branch of the fifth pair of nerves. On examining the patient's teeth, several were found diseased. Five were extracted; at the roots of three of them there existed an abscess. The gums continued sore for some time; but the photophobia was considerably relieved. The patient passed from under the care of Dr. Hays into that of another physician. He heard subsequently that she had entirely recovered; and as no other treatment had been resorted to in her case, excepting covering the eye with a single slip of linen moistened with water, and a shower bath, he believes that he is not in error in referring the cure in this case to the extraction of the diseased teeth.

The last case he shall refer to was that of a young lady from the West. She had been subject to frequent severe attacks of inflammation of the eyes. In July last she suffered from one of these attacks, which was followed by excessive intolerance of light, that no remedy employed seemed in the least to relieve. She was taken to Washington City, where she was pronounced to be incurable by several physicians to whom her case was made known. She was then brought on to Philadelphia, and placed under the care of Dr. H. At his first visit, so great was the intolerance of light, that no satisfactory examination of the eyes could be made. From the imperfect view Dr. H. obtained of them, he ascertained that they were somewhat inflamed, and that a slight opacity of the cornea existed. Several of the young lady's teeth were decayed. Dr. H. directed two to be extracted, but without much relief to the morbid sensibility of the eyes. In a week or ten days, two more of the teeth were extracted from the upper jaw. After a few days the intolerance of light was greatly diminished. The lids were painted with tincture of iodine, and treatment directed, calculated for the relief of the vascularity and opacity of the cornea, and in three months, she so far recovered as to be able to read in a diamond print bible, and bear an ordinary degree of light, when she left for home. Since then the Doctor has not heard from her.

If he is not mistaken in his view of these cases, and he could relate several others of the same character, Dr. Hays believes they very conclusively show that intolerance of light, and other uneasy sensations of the eye, may originate in an irritation of the dental branch of the fifth pair of nerves, resulting from diseased teeth—and the cure of which can only be effected by the removal of the latter. The first two cases appear to prove this incontestably; the symptoms, after resisting all the other means of cure adopted, promptly disappeared upon the extraction of one or more decayed teeth, thus showing the due relation between the effect and its cause. In the last described case, though the intolerance of light was, no doubt, owing to the inflammation of the eye itself, still he conceives that it was in part, at least, kept up by irritation of the dental branch of the fifth pair, from decayed teeth.—*Transactions of the Philadelphia College of Physicians.*

